# Triclosan-Evoked Neurotoxicity Involves NMDAR Subunits with the Specific Role of GluN2A in Caspase-3-Dependent Apoptosis

**DOI:** 10.1007/s12035-018-1083-z

**Published:** 2018-04-19

**Authors:** Konrad A. Szychowski, Agnieszka Wnuk, Joanna Rzemieniec, Małgorzata Kajta, Teresa Leszczyńska, Anna K. Wójtowicz

**Affiliations:** 1 Department of Clinical Biochemistry, University of Opole, Kominka 6a, 45-032 Opole, Poland; 2 Department of Experimental Neuroendocrinology, Institute of Pharmacology, Polish Academy of Sciences, Smetna 12, 31-343 Krakow, Poland; 3 Department of Human Nutrition, Faculty Of Food Technology, University of Agriculture, Balicka 122, 30-149 Krakow, Poland; 4 Department of Animal Biotechnology, Faculty of Animal Sciences, University of Agriculture, Redzina 1B, 30-248 Krakow, Poland

**Keywords:** Triclosan, NMDA, ROS, GluN1, GluN2A, GluN2B

## Abstract

Triclosan (TCS) is an antimicrobial agent that is used extensively in personal care and in sanitising products. A number of studies have shown the presence of TCS in different human tissues such as blood, adipose tissue, the liver, brain as well as in breast milk and urine. *N*-Methyl-*D*-aspartate receptors (NMDARs) are glutamate-gated ion channels that are widely expressed in the central nervous system and which play key roles in excitatory synaptic transmission. There is, however, no data on the involvement of NMDAR subunits in the apoptotic and neurotoxic effects of TCS. Our experiments are the first to show that TCS used at environmentally relevant concentrations evoked NMDA-dependent effects in neocortical neurons in primary cultures, as MK-801, an uncompetitive NMDA receptor antagonist, reduced the levels of TCS-induced ROS production as well as caspase-3 activity and LDH release. TCS caused a decrease in protein expression of all the studied NMDA receptor subunits (GluN1, GluN2A, GluN2B) that were measured at 3, 6 and 24 h post-treatment. However, at 48 h of the experiment, the level of the GluN1 subunit returned to the control level, and the levels of the other subunits showed a tendency to increase. In TCS-treated neocortical cells, protein profiles of NMDAR subunits measured up to 24 h were similar to mRNA expression of GluN1 and GluN2A, but not to GluN2B mRNA. In this study, cells transiently transfected with GluN1, GluN2A or GluN2B siRNA exhibited reduced levels of LDH release, which suggests the involvement of all of the studied NMDAR subunits in the neurotoxic action of TCS. According to our data, GluN1 and GluN2A were mainly responsible for neuronal cell death as evidenced by neutral red uptake, whereas GluN2A was involved in TCS-induced caspase-3-dependent apoptosis. We suggest that TCS-evoked apoptosis and neurotoxicity could be related to transient degradation of NMDAR subunits in mouse neurons. Furthermore, recycling of NMDAR subunits in response to TCS is possible. Because transfections with specific siRNA did not completely abolish the effects of TCS as compared to cells transfected with negative siRNA in this study, other NMDAR-independent mechanisms of TCS action are also possible.

**Electronic supplementary material:**

The online version of this article (10.1007/s12035-018-1083-z) contains supplementary material, which is available to authorized users.

## Introduction

Triclosan (IUPAC name: 5-chloro-2-(2,4-dichlorophenoxy)phenol; CAS number: 3380-34-5) (TCS) is an antimicrobial agent that is commonly used worldwide. Due to both its anti-bacterial and anti-mould properties, it is added to a wide range of personal care and sanitising products as well as to household items or medical devices [1–5]. The main source of TCS in the environment is in personal care products, which contain 0.1% to a maximum of 0.3% TCS [6, 7]. Due to its lipophilicity, TCS can easily pass through biological barriers and can be accumulated in living organisms [6, 8]. A number of studies have revealed the presence of TCS in human tissues, such as in fat, the liver, brain and also in blood or breast milk [9–12]. Alarming concentrations of TCS have been detected in fish (13–88 ng/g) and human (0.23 ng/g) brains [10, 13]. However, only a few papers have examined the TCS mechanism of action in neuronal cells [14–16].

*N*-Methyl-*D*-aspartate receptors (NMDARs) mediate excitatory neuronal transmission. However, despite their physiological functions, a large amount of evidence indicates their involvement in excitotoxicity [17, 18]. NMDARs are assembled as heteromers that differ in subunit composition. To date, seven different subunits have been identified, namely the GluN1 subunit, four distinct GluN2 subunits (GluN2A, GluN2B, GluN2C and GluN2D) and a pair of GluN3 (GluN3A and GluN3B) [18, 19]. Functional NMDA receptors require the assembly of two GluN1 subunits together with two GluN2 subunits or a combination of GluN2 and GluN3 subunits [19, 20]. Di-heteromeric GluN1/GluN2B and GluN1/GluN2A receptors are an important fraction of juvenile and adult NMDARs. Moreover, in the adult central nervous system, particularly in structures such as the hippocampus and cortex, GluN2A and GluN2B are the predominant subunits [21, 22].

Reactive oxygen species (ROS) have a wide spectrum of functions in neuronal cells, where they can be generated as by-products of cellular metabolism, primarily in the mitochondria [23]. ROS and oxidative stress damage are intimately linked to glutamate neurotoxicity, known as excitotoxicity [24]. Excitotoxicity refers to excessive activation of neuronal amino acid receptors. The specific type of excitotoxicity triggered by the amino acid glutamate is the key mechanism implicated in the mediation of neuronal death in many disorders [25].

To date, it has been proved that TCS induces the activity of enzymes involved in ROS metabolism in cells from different organisms such as green ormer (*Haliotis tuberculata*) hemocytes, zebra mussel (*Dreissena polymorpha*) hemocytes, terrestrial snail (*Achatina fulica*), *Daphnia magna* or earthworms (*Eisenia fetida*) [26–31]. However, TCS-stimulated ROS production has been poorly studied in mammalian cells. The only available data refer to the human lung epithelial (A549) cell line, rat embryonal stem cells and mouse neuronal cells [14, 16, 32]. Furthermore, up to today, no studies have been undertaken to investigate the involvement of NMDARs or ROS-dependent excitotoxicity in the nervous system’s response to TCS.

The aim of this study was to determine the involvement of NMDAR subunits in triclosan-evoked apoptosis and neurotoxicity in mouse neocortical neurons in primary cultures. Molecular analyses including mRNA and protein expression measurements as well as siRNA silencing were applied to support biochemical data related to ROS, neutral red uptake and caspase-3 and LDH activities.

## Methods

### Reagents

Neurobasal medium without phenol red, B27-AO supplement and TaqMan probes corresponding to specific genes encoding *Actb* (Mm00607939_s1), *GluN1* (Mm00433800_m1), *GluN2A* (Mm00433802_m1) and *GluN2B* (Mm00433820_m1) were purchased from Life Technologies (Grand Island, NY, USA). Trypsin, charcoal/dextran-treated fetal bovine serum (FBS), L-glutamate, penicillin, streptomycin, *N*-methyl-*D*-aspartate acid (NMDA), staurosporine, triclosan (Irgasan), neutral red uptake assay, 2′,7′-dichlorodihydrofluorescein diacetate (H_2_DCFDA), dizocilpine ((+)-MK801 maleate) and dimethyl sulfoxide (DMSO) were purchased from Sigma-Aldrich (St. Louis, MO, USA). The lactate dehydrogenase-based cytotoxicity detection kit was purchased from Roche Applied Science (Mannheim, Germany). INTERFERin siRNA transfection reagent was purchased from Polyplus-transfection (Illkirch, France). NMDAζ1 siRNA (GluN1) (sc-36082), NMDAε1 siRNA (GluN2A) (sc-36,084), NMDAε2 siRNA (GluN2B) (sc-36086), anti-NMDAζ1 antibody (sc-1467), anti-NMDAε1 antibody (sc-1468), anti-NMDAε2 antibody (sc-1469) and anti-GAPDH antibody (sc-25778) were purchased from Santa Cruz Biotechnology, Inc. (Santa Cruz, CA, USA). Phosphate-buffered saline (PBS) was purchased from BIOMED (Lublin, Poland). Caspase-3 substrate was purchased from Calbiochem (Merck Corporation, Darmstadt, Germany). TCS and other reagents were dissolved in DMSO. The final DMSO concentration in the culture medium was always equal to 0.01%.

### Primary Cultures of Neocortical Neurons

The experiments were performed on primary cultures of mouse neocortical neurons. These cultures were prepared from the foetuses of pregnant female Swiss mice as previously described in Brewer [33] and modified by Szychowski et al. [15]. Brain tissues were collected from the mouse embryos on day 15/16 of gestation. Pregnant females were anesthetised with CO_2_ and killed by cervical dislocation. The animal care protocols were in accordance with official governmental guidelines, and all efforts were made to minimise the number of animals used and their suffering. All procedures were performed in accordance with the National Institutes of Health Guidelines for the Care and Use of Laboratory Animals and were approved by a Bioethics Commission (no. 46/2014) in compliance with Polish law. The brains were removed from the foetuses and the cortical tissues were dissected. The dissected tissue was minced into small pieces and then gently digested with trypsin. Then, the cells were centrifuged and the pellet was suspended in phenol red-free Neurobasal medium supplemented with 5% charcoal/dextran-treated FBS. The cells were plated onto poly-L-ornithine-coated (0.01 mg/mL) multi-well plates. After 2 days, the culture medium was exchanged with Neurobasal medium supplemented with B27-AO (2 μL/mL), glutamine (2 mM), 10 U/mL penicillin and 0.01 mg/mL streptomycin, without FBS which is recommended for primary neuronal cultures [33, 34]. The cells were cultured at a density of 1.8 × 10^5^ cells/cm^2^ for the experiments. The cultures were maintained at 37 °C in a humidified atmosphere containing 5% CO_2_ and were cultivated for 7 days in vitro prior to the experiment. Then, the culture medium was changed prior to treating the cultures with the compound selected for this study.

### siRNA Gene Silencing Procedure

GluN1, GluN2A and GluN2B siRNA was used to inhibit expression of NMDA receptor subunits in mouse neocortical neurons by using a modification of a previously described method [35]. The siRNA was applied for 7 h at a final concentration of 50 nM in antibiotic-free medium containing the siRNA transfection reagent INTERFERin. The cells were plated on 96-well plates for the experiments. Vehicle controls included positive siRNA and negative siRNA containing a scrambled sequence that did not lead to specific degradation of any known cellular mRNA. The culture medium was changed after transfection, and the cells were incubated for 12 h before starting the experiment with 10 μM TCS, 10 μM MK-801 NMDA receptor antagonist and 1 mM L-glutamate NMDA receptor agonist for 24 h. LDH release, neutral red uptake and caspase-3 activity were determined.

### LDH Cytotoxicity Assay

Due to the different aspects measured by cell viability assays, authors have chosen LDH release and neutral red uptake assays. The cytotoxicity detection kit is a colorimetric assay for the quantification of cell death and cell lysis based on the release of lactate dehydrogenase (LDH) from the cytosol of damaged cells into the surrounding medium [36]. An increase in the amount of dead or plasma membrane-damaged cells results in an increase in LDH activity in the culture medium. The cells were plated on 96-well plates for the assays. After 6 and 24 h of treatment with 10 μM TCS, 10 μM MK-801 (NMDA receptor antagonist) or 1 mM L-glutamate (NMDA receptor agonist), 100 μL of the culture supernatants was collected to estimate the LDH and plates with cells were frozen at − 80 °C to measure caspase-3 activity. To measure cytotoxicity, the reaction was stopped after 30 min by adding 1 N HCl and absorbance at a wavelength of 490 nm was measured using the ELISA microplate reader manufactured by Bio-Tek Instruments (Biokom).

### Caspase-3 Activity

Caspase-3 activity was used as a marker of cell apoptosis and was assessed according to Nicholson et al. [37]. For measurement of caspase-3 activity, the cells were plated on 96-well plates and exposed to 10 μM TCS, 10 μM MK-801 or 1 mM L-glutamate for 6 and 24 h. After thawing (− 80 °C), the neurons were lysed using lysis buffer (50 mM HEPES, pH 7.4, 100 mM NaCl, 0.1% CHAPS, 1 mM EDTA, 10% glycerol and 10 mM DTT) in 10 °C for 10 min. The lysates were incubated in caspase-3 substrate Ac-DEVD-pNA at 37 °C. Cells treated with 1 μM staurosporine were used as a positive control (data not shown). After 30 min, absorbance of the lysates at 405 nm was measured using a microplate reader (Bio-Tek ELx800). The amount of colorimetric product was continuously monitored for 120 min. The data were analysed using KCJunior software (Bio-Tek Instruments) and were normalised to absorbance in the vehicle-treated cells.

### Neutral Red Uptake Cytotoxicity Assay

The number of viable cells in experimental conditions was evaluated using the neutral red uptake test. This method is based on the ability of viable cells to incorporate and bind the supravital dye neutral red in the lysosomes. The neutral red uptake cytotoxicity assay is commonly used to study the viability of in vitro cultured primary cells as well as cell lines of diverse origin [38]. After 24 h of exposure to 10 μM TCS, 10 μM MK-801 or 1 mM L-glutamate, the culture medium was removed and the cells were incubated for 2 h in 100 μL Neurobasal medium containing 10% neutral red. Each well was washed with 150 μL PBS and incubated with 100 μL of acidified ethanol solution (50% ethanol, 1% acetic acid, 49% H_2_O) for 10 min at room temperature on a rotating platform. Absorbance was measured at a wavelength of 540 nm using a FilterMax F5 Multi-Mode microplate reader (Molecular Devices, Corp., Sunnyvale, CA, USA).

### ROS Production

The fluorogenic dye 2′,7′-dichlorodihydrofluorescein diacetate (H_2_DCFDA) was used to detect intracellular reactive oxygen species (ROS). After diffusing into the cell, H_2_DCFDA is de-acetylated by cellular esterases into a non-fluorescent compound that is subsequently oxidised by ROS into 2′,7′-dichlorofluorescein (DCF) [39]. We applied 5 μM H_2_DCFDA in order to determine the ability of TCS to induce ROS production in neocortical neurons. We selected 3 and 6 h to study these processes as ROS production caused by NMDAR activation occurs in a short time period. The cells were plated on black-sided, clear-bottomed 96-well plates and exposed to vehicle (control group), 10 μM TCS and L-glutamate for an appropriate time period with or without MK-801 for the ROS measurement. The cells were incubated in H_2_DCFDA in serum-free and phenol red-free Neurobasal medium for 45 min prior to TCS treatment. Before measurement, the culture medium was replaced with fresh Neurobasal medium (FBS free) to remove extracellular residual DCF and H_2_DCFDA to reduce the fluorescence background. Fluorescence was measured after 3 and 6 h of incubating the cells with TCS and tool compounds (5% CO2 at 37 °C). DCF fluorescence was detected using a microplate reader (FilterMax F5) at maximum excitation and emission spectra of 485 and 535 nm, respectively. Cells treated with 0.3% hydrogen superoxide were used as a positive control (results not shown). The interaction between TCS and H_2_DCFDA was tested under cell-free conditions prior to the experiments (results not shown) based on concerns that were raised about the H_2_DCFDA assay as previously described by Szychowski et al. [40, 41].

### Real-time PCR Analysis of mRNAs Specific to Genes Encoding *GluN1*, *GluN2A* and *GluN2B*

For the real-time PCR assay, neurons were seeded on poly-L-ornithine-coated 6-well plates and initially cultured for 7 days. After 3 or 6 h of exposure to 10 μM TCS, samples were collected and total RNA was extracted from neocortical neurons using a Qiagen RNeasy mini kit according to the manufacturer’s protocol and based on a previously described method [35]. The quality and quantity of the RNA was determined spectrophotometrically at 260 and 280 nm (ND/1000 U*V*/Vis; Thermo Fisher NanoDrop, USA). Two-step real-time RT-PCR was conducted with both the reverse transcription (RT) reaction and the quantitative polymerase chain reaction (qPCR) run using the CFX96 Real Time System (Bio-Rad, USA). The RT reaction was performed at a final volume of 20 μL with 300 ng of RNA (as a cDNA template) using the cDNA reverse transcription kit according to the manufacturer’s protocol. Products from the RT reaction were amplified using the TaqMan Gene Expression Master Mix (Life Technologies Applied Biosystems, USA) kit with TaqMan probes as primers for specific genes encoding *Actb*, *GluN1*, *GluN2A* and *GluN2B.* Amplification was conducted in a total volume of 20 μL containing the 1x TaqMan Gene Expression Master Mix and 1 μL of the RT product, which was used as the PCR template. Standard qPCR procedures were performed as follows: 2 min at 50 °C and 10 min at 95 °C, followed by 40 cycles of 15 s at 95 °C and 1 min at 60 °C. The threshold value (Ct) for each sample was set during the exponential phase, and the delta Δ Ct method was used for data analysis. To study the gene expression levels, five candidate reference genes (*Actb*, *Gapdh*, *B2m*, *Hprt*, *18S*) were selected and validated. To evaluate the reference gene expression, RefFinder web-based comprehensive tool has been used [42]. RefFinder integrates major computational programs (geNorm, Normfinder, BestKeeper and the comparative delta Ct method) to compare and rank candidate reference genes [43–46]. In our study, NormFinder, BestKeeper and delta Ct recommended *Actb* as the most stable reference genes according to 3 and 6 h exposure to 10 μM TCS (supplementary data).

### Western Blot Analysis

For the Western blot assay, neurons were seeded on poly-L-ornithine-coated 6-well plates and were initially cultured for 7 days. After 1, 3, 6, 24 or 48 h of exposure to 10 μM TCS, Western blot samples were collected and GluN1, GluN2A and GluN2B protein expression levels were measured. For immunoblotting, the cells were lysed in 100 μL of ice-cold lysis buffer containing 100 mM NaCl, 50 mM Tris HCl (pH 7.5), 0.5% Na-deoxycholate, 0.5% Nonidet NP-40 and 0.5% SDS. Then, the lysates were sonicated and clarified by centrifugation at 15,000×*g* at 4 °C for 20 min and the supernatant was collected and stored at − 80 °C until analysis. Protein concentrations in the supernatants were determined using the Bradford method [47] with bovine serum albumin (BSA) as the standard. From the whole cell lysate, 40 μg of total protein was reconstituted in the appropriate amount of sample buffer, which consisted of 125 mM Tris (pH 6.8), 4% SDS, 25% glycerol, 4 mM EDTA, 20 mM DTT and 0.01% bromophenol blue. Samples were separated by 7.5% SDS-polyacrylamide gel electrophoresis in a Bio-Rad Mini-Protean II Electrophoresis Cell, and the proteins were then transferred to nitrocellulose membranes using a Bio-Rad Mini Trans-Blot apparatus. Following the transfer, the membranes were washed and nonspecific binding sites were blocked with 5% dried milk and 0.2% Tween 20 in 0.02 M TBS for 2 h. Then, the membranes were incubated overnight with anti-GluN1, anti-GluN2A and anti-GluN2B antibodies diluted at 1:250 in TBS/Tween at 4 °C. Following incubation with the primary antibodies, the membranes were washed with TBS and 0.02% Tween 20 and then incubated for 2 h with horseradish peroxidase-conjugated secondary antibodies diluted to 1:1000 in TBS/Tween. To control for the amount of protein that was loaded onto the gel, we used an anti-GAPDH antibody diluted at 1:1000 in TBS/Tween (secondary antibody diluted at 1:5000 in TBS/Tween). Signals were detected by chemiluminescence (ECL) using a Western blotting luminol reagent and visualised with the use of PhosphorImager FujiLas 4000.

### Statistical Analysis

The data were presented as means ± SD of three independent experiments. Each treatment was repeated eight times (*n* = 8) and measured in triplicate; thus, the total number of replicates was 24. The average of the triplicate samples was used for the statistical analyses. Statistical analysis was performed on the original results. Considering the different data from the measurement of fluorescence or absorbance, the results were presented as a percentage of the controls. The data were analysed via one-way analysis of variance (ANOVA) followed by Tukey’s multiple comparison procedure in STATISTICA 10 software (*p < 0.05, **p < 0.01 and ***p < 0.001 vs. the control. ###*p* < 0.001 vs. cells treated with TCS alone).

## Results

### TCS-Induced Excitotoxicity

#### TCS-Induced ROS Production

**Fig. 1:**
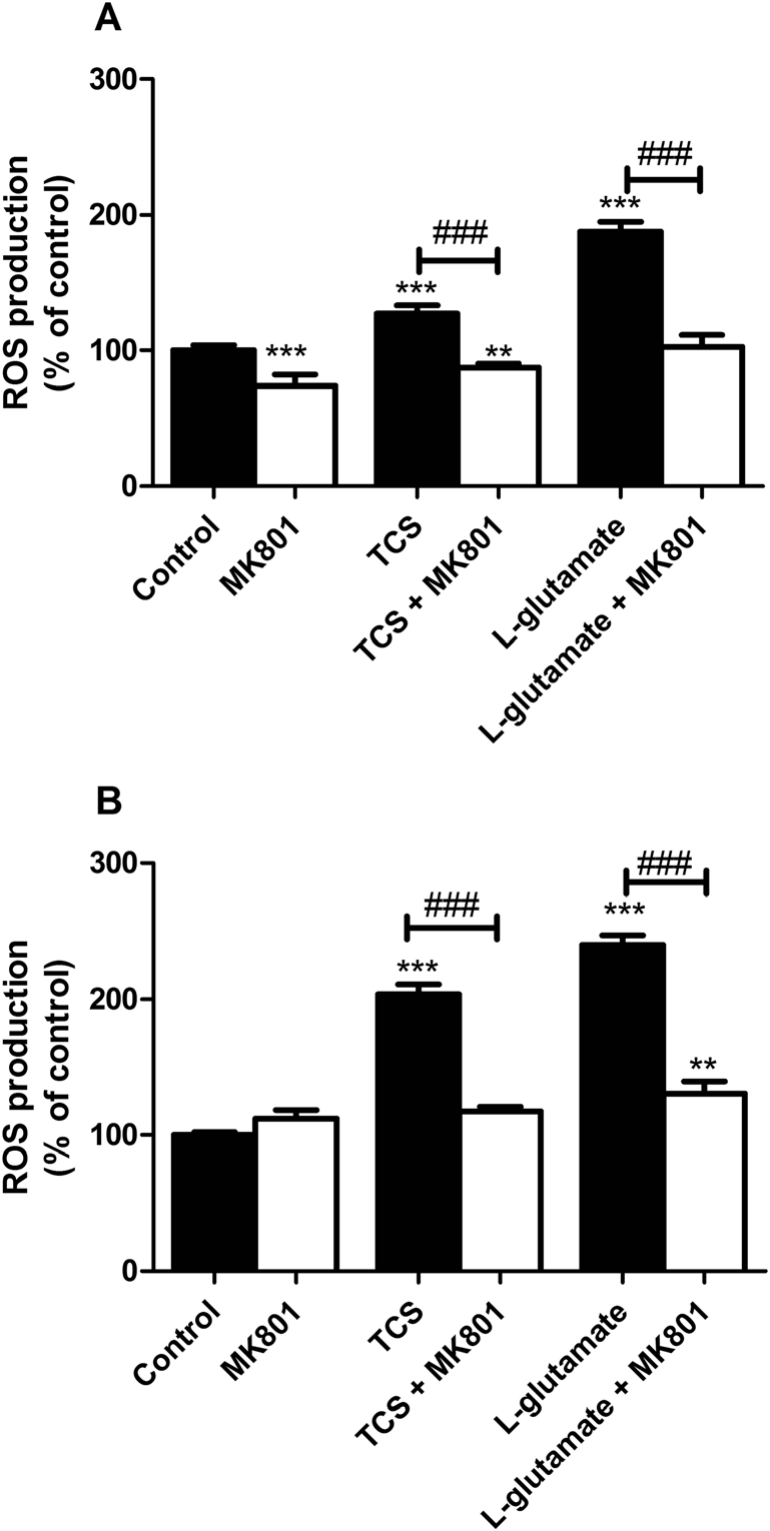
Effect of 10 μM TCS on ROS production after 3 (**a**) and 6 (**b**) h. The addition of 10 μM MK-801 reduced the effect of TCS. The data are expressed as means ± SD of three independent experiments, each of which consisted of eight replicates per treatment group. **p < 0.01; ***p < 0.001 vs. the vehicle control. ###*p* < 0.001 vs. cells treated with TCS alone or cells treated with L-glutamate alone

Following 3 h of exposure, 10 μM TCS increased ROS production by 27% in the neurons as compared to the controls. The presence of 10 μM MK-801 alone reduced the production of ROS below the control level (decrease by 26.06%). Co-treatment with 10 μM MK-801 and 10 μM TCS decreased TCS-induced ROS production as compared to the control (decrease by 12.52%). L-Glutamate used as a positive control increased ROS production by 87.58% in the neurons as compared to the controls. The presence of MK-801 decreased the production of ROS induced by L-glutamate, ROS production back to control levels (Fig. 1a).

Following 6 h of exposure to 10 μM TCS, TCS increased ROS production in the neurons as compared to the controls by 103.57%. The presence of 10 μM MK-801 decreased the production of ROS induced by TCS, ROS production back to control levels. L-Glutamate increased ROS production by 140.20% in the neurons as compared to the controls. The presence of MK-801 decreased the production of ROS induced by L-glutamate by 109.62% (Fig. 1b).

#### TCS-Induced LDH Release

**Fig. 2:**
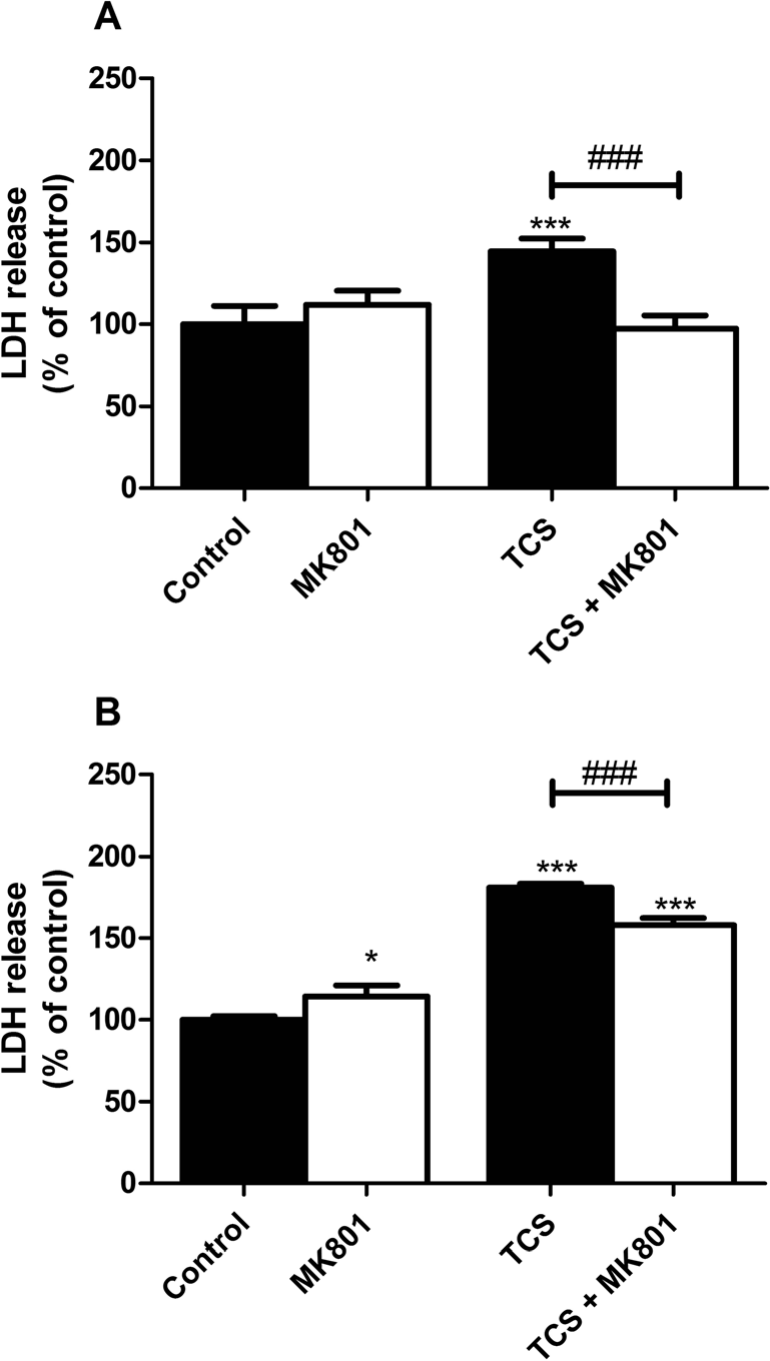
Effect of 10 μM TCS on LDH release after 6 (**a**) and 24 (**b**) h. The addition of 10 μM MK-801 reduced the effect of TCS. The data are expressed as means ± SD of three independent experiments, each of which consisted of eight replicates per treatment group. *p < 0.05; ***p < 0.001 vs. the vehicle control. ###*p* < 0.001 vs. cells treated with TCS alone

Following 6 h of exposure to 10 μM TCS, TCS increased LDH release in the neurons as compared to the controls (increases by 44.41%). Cell co-treatment with 10 μM MK-801 and 10 μM TCS decreased TCS-induced LDH release to the control level (Fig. 2a).

Following 24 h of exposure to 10 μM TCS, TCS increased LDH release in the neurons as compared to the controls (increases by 81.12%). Cell co-treatment with MK-801 and 10 μM TCS decreased TCS-induced LDH release by 23.03% (Fig. 2b).

#### TCS-Induced Caspase-3 Activity

**Fig. 3:**
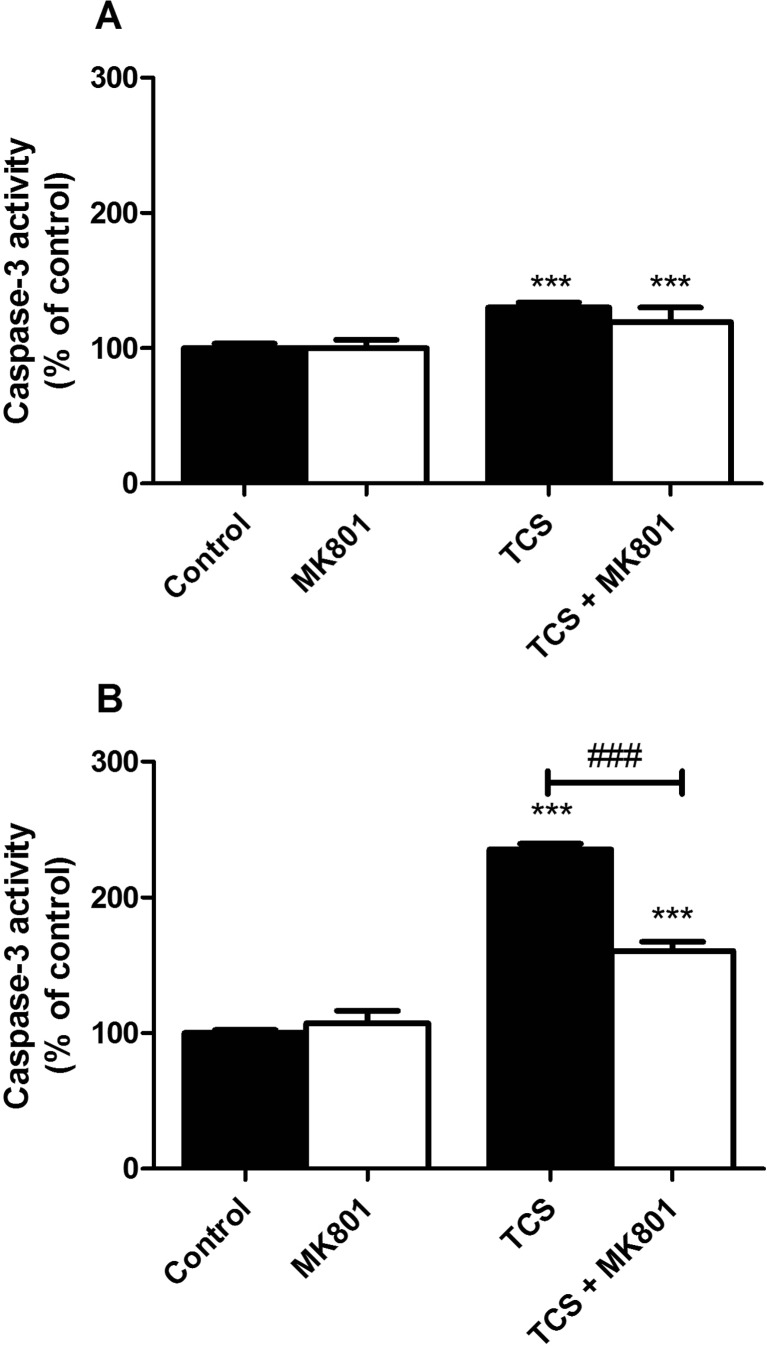
Effect of 10 μM TCS on caspase-3 activity after 6 (**a**) and 24 (**b**) h. The addition of 10 μM MK-801 reduced the effect of TCS. The data are expressed as means ± SD of three independent experiments, each of which consisted of eight replicates per treatment group. ***p < 0.001 vs. the vehicle control. ###*p* < 0.001 vs. cells treated with TCS alone

Following 6 h of exposure to 10 μM TCS, TCS increased caspase-3 activity in the neurons as compared to the controls (increases by 30.10%). In cells co-treated with 10 μM MK-801 and 10 μM TCS, we observed no decreased TCS-induced caspase-3 activity (Fig. 3a).

Following 24 h of exposure to 10 μM TCS, TCS increased caspase-3 activity in the neurons as compared to the controls (increases by 135.25%). In cells co-treated with MK-801 and 10 μM TCS, we observed a decrease in TCS-induced caspase-3 activity by 74.73% (Fig. 3b).

### Real-time PCR Analysis of mRNAs Specific to Genes Encoding *GluN1*, *GluN2A* and *GluN2B*

**Fig. 4:**
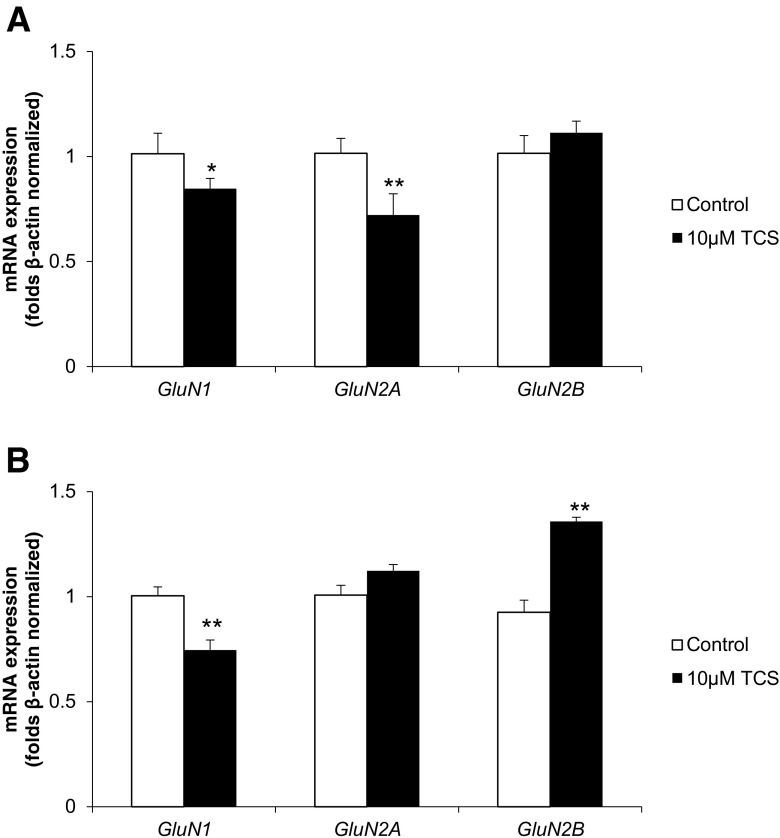
Effect of 10 μM TCS on mRNA expression of *GluN1*, *GluN2A* and *GluN2B* after 3 (**a**) and 6 (**b**) h of exposure. mRNA expression was normalised to β-actin. The data are expressed as means ± SD of three independent experiments, each of which consisted of eight replicates per treatment group. *p < 0.05; **p < 0.01 vs. the vehicle control

Following 3 h of exposure to 10 μM TCS, the neocortical neurons showed a decrease in their expression of *GluN1* and *GluN2A* mRNA as compared to the vehicle control (decrease of 15.27 and 27.81%, respectively) (Fig. 4a).

Following 6 h of exposure to 10 μM TCS, we observed a decrease in the expression of *GluN1* mRNA as compared to the vehicle control (decrease of 25.39%). However, in the same time period, 10 μM TCS increase in the expression *GluN2B* mRNA compared to the vehicle control (increase of 35.74%) (Fig. 4b).

### Effects of TCS on Expression of the GluN1, GluN2A and GluN2B Protein

**Fig. 5:**
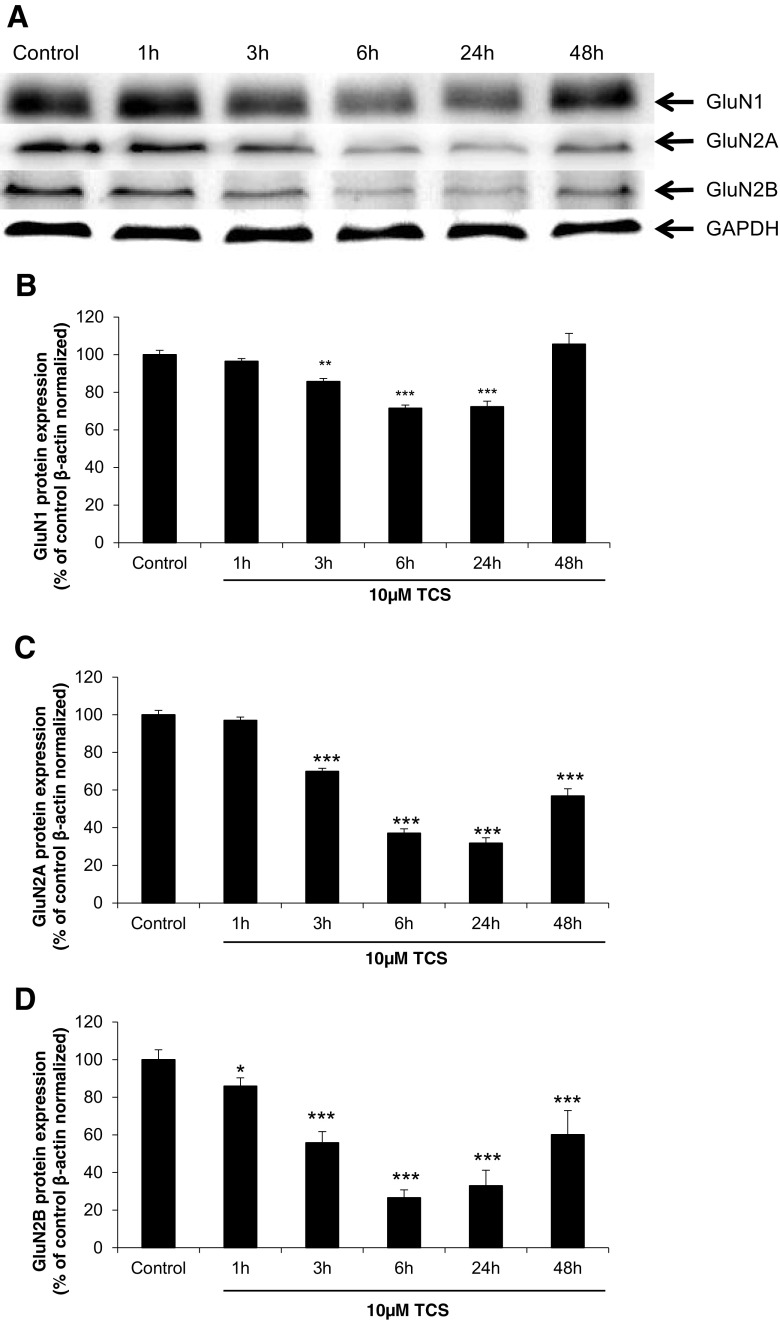
Representative Western blot of GluN1, GluN2A and GluN2B protein levels from neocortical neurons treated with 10 μM TCS after 1, 3, 6, 24 or 48 h (**a**). Protein bands were quantified by densitometry. The results are shown as the percentage of GluN1, GluN2A and GluN2B proteins relative to the control. Each column represents the mean ± SD of three independent experiments (**b**–**d**). The blots were stripped and reprobed with the anti-GAPDH antibody to control for the amounts of protein loaded onto the gel. **p < 0.01, ***p < 0.001 vs. the vehicle control

Immunoblot analyses quantified by densitometry showed that in neurons treated with 10 μM TCS for 3, 6 and 24 h, the level of the GluN1 protein was decreased as compared to the vehicle control cells by 14.31, 28.47 and 27.76%, respectively. However, after 48 h, expression of the GluN1 protein returned to the control level. The decrease in GluN2A protein expression was observed after 3, 6, 24 and 48 h of exposure to 10 μM TCS (compared to the control, 30.07, 62.96, 68.14 and 43.16%, respectively). GluN2B protein expression started to decrease after 1 h (by 14.06% as compared to the control) and continued to decrease at 3, 6, 24 and 48 h (decreased by 44.12, 73.44, 67.02 and 39.88%, respectively) (Fig. 5).

### Silencing of NMDAR Subunits in TCS Toxicity

#### Involvement of NMDARs in TCS-Induced Neutral Red Uptake Assay

**Fig. 6:**
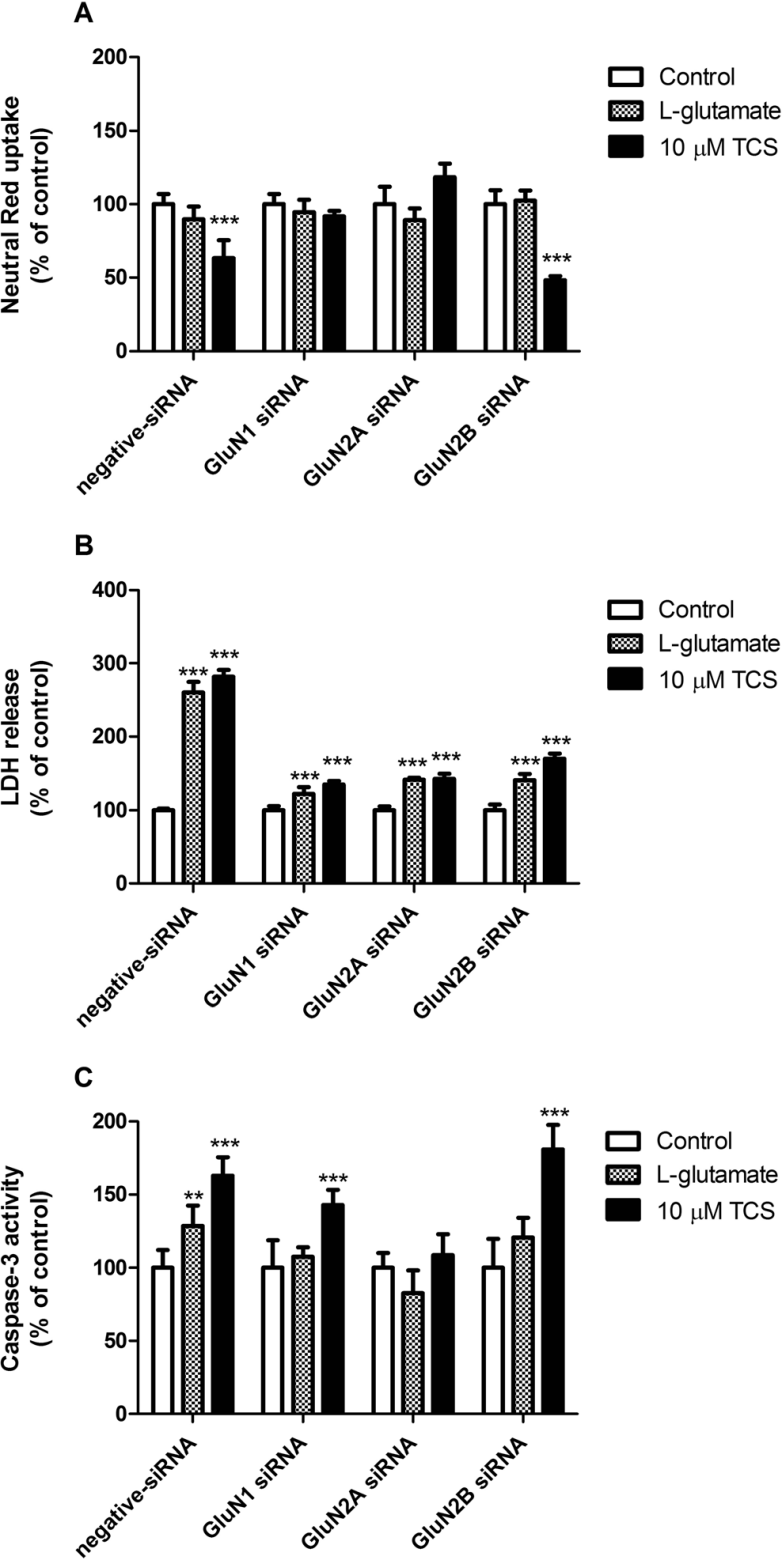
Effects of 10 μM TCS, 10 μM MK-801 or 1 mM L-glutamate on neutral red uptake (**a**), release of LDH (**b**) and caspase-3 activity (**c**) on negative siRNA and NMDAR subunit (GluN1, GluN2A and GluN2B) siRNA-transfected cells after 24 h of exposure. Data are expressed as means ± SD of three independent experiments, each of which comprised eight replicates per treatment group. **p < 0.01; ***p < 0.001 vs. the vehicle control

Following 24 h of exposure to 10 μM concentrations of TCS, neurons transfected with negative siRNA showed a decrease in neutral red uptake as compared to the vehicle control (decrease by 36.72%). In neurons transfected with GluN2B siRNA, a similar decrease was observed in neutral red uptake as compared to the vehicle control (decrease by 51.75%). In neurons transfected with GluN1 or GluN2A siRNA, no changes were observed (Fig. 6a).

#### Involvement of NMDARs in TCS-Induced LDH Release

Following 24 h of exposure to 10 μM concentrations of TCS, neurons transfected with negative siRNA showed an increase in LDH release as compared to the vehicle control by 181.79%. After transfection with GluN1, GluN2A or GluN2B siRNA, we observed an increase in LDH release as compared to the control by 34.42, 42.24 and 69.99%, respectively (Fig. 6b).

#### Involvement of NMDARs in TCS-Induced Caspase-3 Activity

Following 24 h of exposure to 10 μM TCS, neurons transfected with negative siRNA showed enhanced caspase-3 activity as compared to the vehicle controls by 62.99%. In cells transfected with GluN1 and GluN2B siRNA, TCS activated caspase-3 by 42.75 and 80.99%, respectively, as compared to the controls (Fig. 6c).

## Discussion

Our experiments are the first to show that TCS used at environmentally relevant concentrations activated caspase-3 and stimulated ROS production as well as LDH release in neocortical neurons in primary cultures. These effects were NMDA-dependent since MK-801, an uncompetitive NMDA receptor antagonist, reduced the levels of TCS-induced caspase-3 activity and LDH release at 6 and 24 h post-treatment. As for ROS formation, at 3 h of the experiment, MK-801 reduced its control level, but at 6 h of the experiment, the control level of ROS remained unchanged, thus supporting the specific action of the NMDA antagonist on neocortical neurons that were treated with TCS for 6 h. It has been shown that excitotoxicity depends on NMDAR activation, which results in the formation of large amounts of ROS such as the superoxide anion, hydrogen peroxide, nitric oxide or hydroxyl radical [48–50]. To date, TCS-stimulated ROS production has been poorly studied in mammalian cells. The only available data refer to the A549 cell line, rat embryonal stem cells and mouse neuronal cells [14, 16, 32].

In this study, we demonstrated that TCS-stimulated ROS formation was accompanied by NMDA-dependent activation of caspase-3. According to our data, 24 h exposure to MK-801 decreased TCS-evoked enzyme activity, which confirms the involvement of NMDARs in the apoptotic action of TCS. High levels of ROS are known to damage the mitochondria and to initiate the intrinsic apoptosis pathway in an NMDA receptor-dependent manner [51–53]. To date, Szychowski et al. (2015) have showed that TCS activated caspase-9 in mouse neurons, which suggests an intrinsic apoptosis pathway [15]. In the present study, NMDAR mediated not only the induction of ROS formation and activation of caspase-3 in response to TCS but also LDH release. Our experiments showed that after 6 and 24 h of exposure, the MK-801 decreased the TCS-induced LDH release.

Quantitative PCR (qPCR) and Western blot analyses were conducted to confirm involvement of the NMDA receptor in the apoptotic and neurotoxic action of TCS. Our study is the first to show that TCS caused a decrease in protein expression of all of the studied NMDA receptor subunits (GluN1, GluN2A, GluN2B) that were measured at 3, 6 and 24 h post-treatment. However, at 48 h of the experiment, the level of the GluN1 subunit returned to the control level, and the levels of the other subunits, GluN2A and GluN2B, showed a tendency to increase. In TCS-treated neocortical cells, the protein profiles of NMDAR subunits measured up to 24 h were similar to the mRNA expression of GluN1 and GluN2A, but not GluN2B mRNA. We suggest that the differences between mRNA and the protein profiles of the NMDA subunits were due to subunit-specific regulation in response to TCS.

There has been no other study on TCS-evoked changes in mRNA and protein expression of NMDA receptor subunits in order to be able to compare them to our results. In the environment, TCS is converted to dioxins such as 2,8-DCDD, 2,3,7-TCDD, 1,2,8-TriCDD and 1,2,3,8-TCDD [54, 55]. Because of the structural similarity of TCS and dioxins, one may compare their effects with respect to the expression of NMDA receptor subunits in brain neurons. Similarly to the effect of TCS in our study, Hood et al. (2006) showed an increase in mRNA expression of the *GluN2B* subunit in the brain tissue of rats that were prenatally exposed to TCDD [56]. As was observed in our study in response to TCS, Nayyar et al. (2003) detected reduced mRNA and protein levels of GluN1 in embryonic mouse neurons treated with TCDD [57]. Enhanced protein levels of the GluN2A and GluN2B subunits, as demonstrated by Cho et al. (2002) in TCDD-treated rat embryonic cortical neurons, correspond to some extent to the effects of TCS at 48 h of exposure as were observed in our study [58].

Specific siRNA-mediated silencing was applied to verify the involvement of individual NMDAR subunits in the apoptotic and neurotoxic action of TCS. According to our data, cells transiently transfected with GluN1, GluN2A or GluN2B siRNA exhibited reduced levels of LDH release, which suggests the involvement of all of the studied NMDAR subunits in the neurotoxic action of TCS. It has been documented that NMDAR subunits have various physiological functions in neuronal cells. GluN2A-containing synaptically located NMDARs have been postulated to activate pro-survival signalling pathways, while GluN2B-containing primarily extrasynaptic NMDARs trigger neuronal death signalling [59, 60]. Because transfections with specific siRNA did not completely abolish the effects of TCS as compared to cells transfected with negative siRNA in our study, other NMDAR-independent mechanisms of TCS action are also possible. In addition, we demonstrated that GluN1 and GluN2A are mainly responsible for neuronal cell death as evidenced by neutral red uptake. We also provided evidence that TCS-induced apoptosis of neuronal cells is a GluN2A-dependent process. Numerous studies have showed that endocytosis of NMDARs is regulated by synaptic activity and receptor activation [61]. NMDARs that are removed from the synapses may either be degraded in the endosomes or recycled [62].

We suggest that the TCS-evoked apoptosis and neurotoxicity that is accompanied by a decrease in protein expression of GluN1, GluN2A or GluN2B as observed from 3 to 24 h of the experiment could be related to transient degradation of NMDAR subunits in mouse neurons. Furthermore, recycling of NMDAR subunits in response to TCS is possible since the expression of GluN1 was normalised and the expression of GluN2A and GluN2B showed a tendency to increase at 48 h of exposure.

## Conclusions

Our experiments are the first to show that TCS used at environmentally relevant concentrations evoked NMDA-dependent apoptosis and neurotoxicity. Triclosan-evoked neurotoxicity involved all studied NMDAR subunits, with the particular role of GluN2A in caspase-3-dependent apoptosis. TCS also disrupted mRNA and protein expression of GluN1, GluN2A and GluN2B in mouse neurons in primary cultures. We postulate that TCS-induced apoptosis and neurotoxicity is related to transient degradation of NMDAR subunits; however, other NMDAR-independent mechanisms of TCS action are possible since the silencing of specific NMDAR subunits did not completely abolish the effects of TCS.

## Electronic Supplementary Material

Supplementary data Evaluation of reference genes using RefFinder web-based tool. Computational algorithms (geNorm, NormFinder, BestKeeper and delta Ct) were used to evaluate and identify suitable reference genes. In our study, NormFinder, BestKeeper and delta Ct recommended β-actin as the most stable reference genes according to 3 h (panel A) and 6 h (panel B) exposure to 10 μM TCS. (XLSX 36 kb)

## Abbreviations

AhR: Aryl hydrocarbon receptor
DMSO: Dimethyl sulfoxide
FBS: Fetal bovine serum
H_2_DCFDA: 2′,7′-Dichlorodihydrofluorescein diacetate
LDH: Lactate dehydrogenase
NAC: N-Acetyl-L-cysteine
PBS: Phosphate-buffered saline
ROS: Reactive oxygen species
TCS: Triclosan, 5-Chloro-2-(2,4-dichlorophenoxy) phenol
MK-801: Dizocilpine, (5S,10R)-(+)-5-Methyl-10,11-dihydro-5H-dibenzo[a,d]cyclohepten-5,10-imine maleate
NMDARs: *N*-Methyl-*D*-aspartate receptors
